# Exosomal circRNA in Digestive System Tumors: The Main Player or Coadjuvants?

**DOI:** 10.3389/fonc.2021.614462

**Published:** 2021-06-24

**Authors:** Haoying Wang, Xi Zeng, Ya Zheng, Yuping Wang, Yongning Zhou

**Affiliations:** ^1^ Department of Gastroenterology, The First Hospital of Lanzhou University, Lanzhou, China; ^2^ Key Laboratory for Gastrointestinal Diseases of Gansu Province, Lanzhou University, Lanzhou, China

**Keywords:** exosome, circRNA, digestive system tumors, gastrointestinal tumors, bioinformatics

## Abstract

Exosomes are a type of extracellular microvesicles with a diameter of 40–160 nm. Circular RNA (circRNA) is a type of closed circular RNA molecule that is highly conserved in evolution. Exosomal circRNA plays a vital role in the proliferation, invasion, migration, and drug resistance of digestive system tumors. In this study, we used The Cancer Genome Atlas (TCGA) database, UALCAN, Python crawler, miRTargetLink Human, Database for Annotation, Visualization, and Integrated Discovery (DAVID), micBioinformatic online tool, and Cytoscape software (3.7.1). The results showed that circ-RanGAP1 in gastric cancer, circUHRF1 in hepatocellular carcinoma, and circFMN2 in colorectal cancer regulate the malignant behavior of tumors and affect the expression of their host gene through sponging miR-877-3p, miR-449c-5p, and miR-1182, respectively. Twenty exosomal circRNAs regulate 6,570 target genes through sponging 23 miRNAs. Firstly, 270 of those target genes are regulated by two or more miRNAs, which are highly correlated with 83 tumor-related pathways and six Kyoto Encyclopedia of Genes and Genomes pathways. Secondly, 1,146 target genes were significantly differentially expressed in corresponding digestive system tumors, and functional enrichment analysis revealed that 78 of those were involved in 20 cancer-related pathways. In short, the bioinformatics analysis showed that these exosomal circRNAs are stably expressed in body fluids, and regulate the occurrence and development of gastric cancer, hepatocellular carcinoma, colorectal cancer, and other digestive system tumors through sponging miRNAs. Exosomal circRNAs may be used as biomarkers for the diagnosis of disease and identification of effective therapeutic targets in the future, as well as improve the prognosis of patients with digestive system tumors.

## Introduction

Five of the top 10 tumors with the highest mortality rate worldwide are tumors of the digestive system, including esophageal, gastric, colorectal, hepatocellular, and pancreatic cancers (1). At the same time, esophageal, hepatocellular, gastric, and colorectal cancers rank among the top 10 tumor diseases with the most severe morbidity (1). Hence, the digestive system tumors pose a serious threat to human health worldwide.

In recent years, a large number of genomics studies have investigated the underlying mechanism of malignant tumors. The most common changes are observed in the tumor and immune microenvironment, post-transcriptional modifications (e.g., DNA methylation), and abnormalities in mesenchymal and tumor stem cells (2–6). However, in recent years, an increasing number of studies have shown that are involved in a variety of tumor biological processes in digestive system tumors (7, 8).

Exosomes are a type of extracellular microbubbles with a diameter of 40–160 nm; they are wrapped by a lipid bilayer membrane and secreted by eukaryotic cells (9). Initially, exosomes were considered wastes of cell metabolism (10). However, with the advancement of biological technology, researches gradually realized that exosomes are involved in a widespread mode of intercellular communication that regulates Alzheimer’s disease, diabetes, tumors, and many other diseases (7, 11, 12). Exosomes can promote tumor epithelial–mesenchymal transition, proliferation, invasion, migration, and inhibit tumor cell apoptosis by transporting a variety of proteins, non-coding RNA, metabolites, and lipids (13). In recent years, the potential of exosomes as targets for the diagnosis and treatment of tumors has received extensive research attention, especially with regard to digestive system tumors.

Circular RNA (circRNA) is a type of closed circular RNA molecule, without a 5’ cap structure and 3’ poly-A tail, that is highly conserved in evolution (14). Similar to exosomes, circRNA was initially considered to be a non-functional mis-splicing body. It was later discovered that it plays an irreplaceable role in a variety of biological processes, particularly tumor progression (15). Numerous studies have shown that circRNA acts on remote tissues and cells by being wrapped by exosomes. It has the ability to regulate various signal pathways, thereby promoting the progression of malignant tumors (16, 17).

In this study, we collected experimentally verified exosomal circRNAs from five types of digestive system tumors (i.e., esophageal, gastric, colorectal, liver, and pancreatic cancers), and investigated the biological functions of these circRNAs and their sponging of miRNAs and target genes. Our results showed that exosomal circRNA participated in a complex competing endogenous RNA network, and regulated the occurrence and development of digestive system tumors. They are expected to be used as important diagnostic and treatment markers for digestive system tumors in the future.

## Materials and Methods

### Search for Exosomal circRNAs

Research literature related to exosomal circRNA and digestive system tumors (published prior to August 2020) was retrieved from the PubMed database. The keywords used are as follows: exosomal, circRNA, circular RNA, hsa_circ, esophageal, gastric, hepatocellular, colorectal, pancreatic, cancer, carcinoma, tumor, and neoplasm. Only studies investigating exosomal circRNA through western blotting, reverse-transcription polymerase chain reaction, and cell phenotyping or animal experiments were included in the present analysis. There were no language or other restrictions. All circRNAs in the literature are derived from the detection of clinical samples.

### Expression Levels of the Host Gene of Exosomal circRNA

The UALCAN online tool (http://ualcan.path.uab.edu/index.html) was used to analyze the expression of the host gene of exosomal circRNA (18). The background data of this website are derived from TCGA, which is committed to providing global cancer researchers with extensive and reliable information on the expression of human tumor-related genes, miRNAs and proteins (18). We used Python crawler (https://www.python.org/) to download website data.

### Prediction and Enrichment of miRNA Target Genes

The miRTargetLink Human (https://ccb-web.cs.uni-saarland.de/mirtargetlink/) online tool was used to analyze the target genes of miRNA and create a miRNA-mRNA interaction network (19). The miRTargetLink Human offers detailed information on human microRNA-mRNA interactions in the form of interactive interaction networks (19). Therefore, all the predicted target genes and subsequent data analysis in this study are all based on human-derived genes. The Database for Annotation, Visualization, and Integrated Discovery (DAVID) website (https://david.ncifcrf.gov/) was used to perform Kyoto Encyclopedia of Genes and Genomes (KEGG) analysis and Gene Ontology (GO) analysis of miRNA target genes. The micBioinformatic (http://www.bioinformatics.com.cn/) online tool was used to create graphs for the KEGG and GO analyses (20, 21). **S1**–**S7** shows target genes with fold change >2 or <0.05, and p<0.05.

### Competing Endogenous RNA Network in Digestive System Tumors

The Cytoscape (Version 3.7.1, https://cytoscape.org/) tool was used to construct the circRNA-miRNA-mRNA interaction network.

## Results

**Figure 1:**
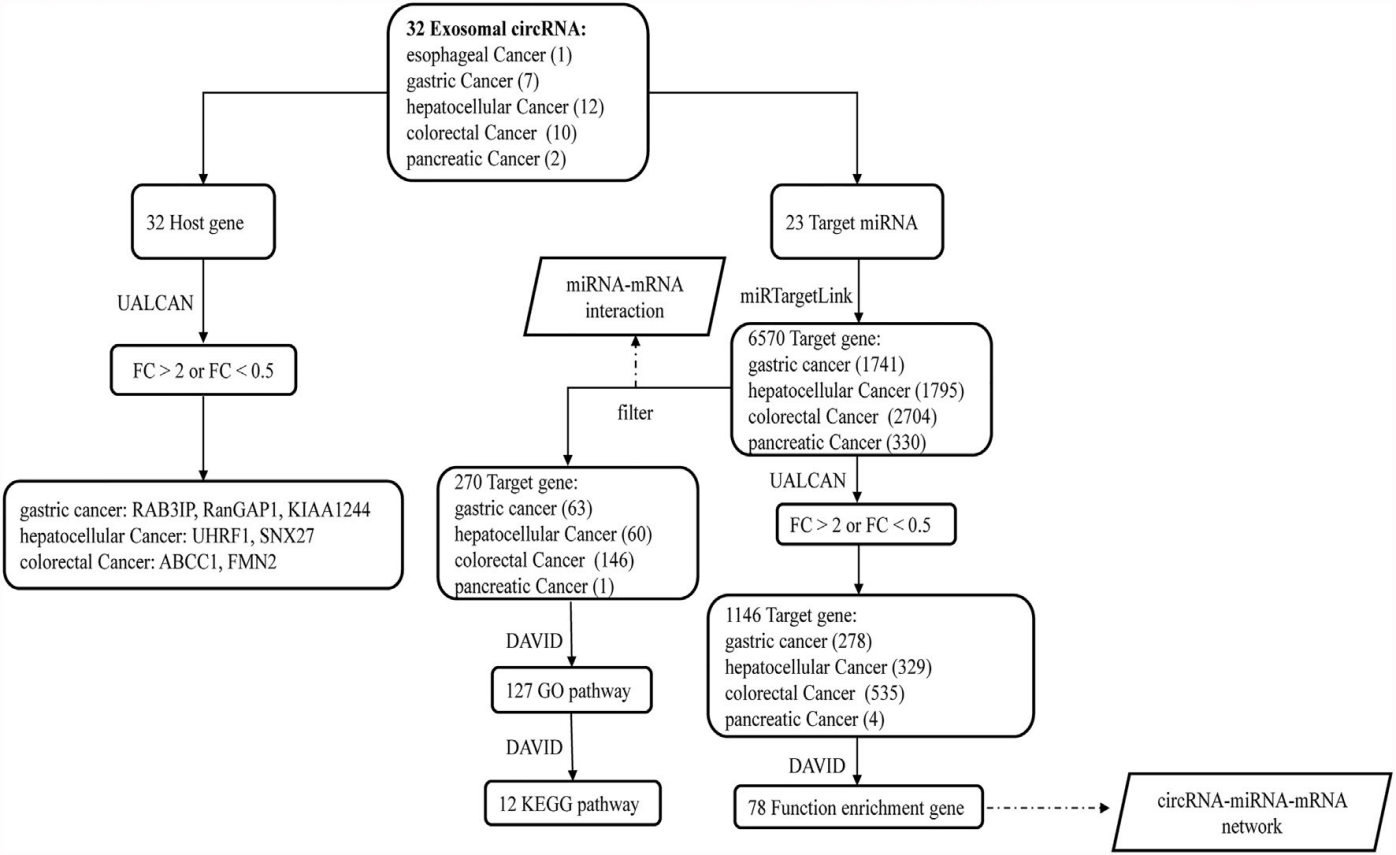
The flow diagram of the study. After obtaining 32 exosomal circRNAs from the literature, we further analyzed their 32 host genes and 23 target miRNAs. Among the 32 host genes, 7 host genes of circRNAs were significantly differentially expressed in the corresponding tumors. Two different methods were used to analyze the downstream target genes and related functions of 23 miRNAs.

We retrieved 30 research articles. From the analysis, we identified 32 exosomal circRNAs related to digestive system tumors, including one for esophageal cancer, seven for gastric cancer, 12 for hepatocellular carcinoma, 10 for colorectal cancer, and two for pancreatic cancer (**Table 1**) (22–51). Among these, 26 molecules were upregulated, six molecules were downregulated, and 20 molecules sponged miRNA (**Table 1**). We separately analyzed the host gene and target miRNA of the 32 exosomal circRNAs. Two different strategies were used to analyze the target gene of miRNA. Finally, the circRNA-miRNA-mRNA interaction network was constructed based on the key molecules obtained. **Figure 1** shows the flow diagram of the study.

**Table 1:** Description of circRNAs in digestive system tumors.

Type of Cancer	CircRNA	Expression	Function	Host gene	Expression	Sponged miRNA	Mechanism	Clinical (Biomarker)	Target Gene	Ref
Esophageal Cancer	hsa_circ_0001946	Down	TS	CDR1	Down^*^			Diagnostic		(22)
Gastric Cancer	hsa_circ_0000936	Up	Oncogene	SHKBP1	Up	miR-582-3p	CeRNA	Diagnostic, Prognosis	HUR/VEGF, HSP90	(23)
	hsa_circ_0000419	Down	TS	RAB3IP	Up			Diagnostic		(24)
	hsa_circ_0065149	Down	TS	SETD2	Up			Prognosis		(25)
	hsa_circ_0063526	Up	Oncogene	RanGAP1	Up	miR-877-3p	CeRNA	Prognosis	VEGFA	(26)
	hsa_circ_0004771	Up	Oncogene	NRIP1	Up	miR-149-5p	CeRNA	Prognosis	AKT1/mTOR	(27)
	hsa_circ_0010522	Up	Oncogene	RAP1GAP	Down^*^	miR-133	CeRNA		PRDM16	(28)
	hsa_circ_0130810	Down	TS	KIAA1244	Up			Prognosis		(29)
Hepatocellular Cancer	hsa_circ_0048677	Up	Oncogene	UHRF1	Up	miR-449c-5p	CeRNA	Resistance to anti-PD1	TIM-3	(30)
	hsa_circ_0004001	Up	Oncogene	CLK1	Up			Prognosis		(31)
	hsa_circ_0004123	Up	Oncogene	ETV6	Up			Prognosis		(31)
	hsa_circ_0075792	Up	Oncogene	KDM1B	Up			Prognosis		(31)
	hsa_circ_0017252	Up	Oncogene	AKT3	Down			Prognosis		(32)
	has_circ_0039411	Up	Oncogene	MMP2	Up	miR-136-5p	CeRNA	Prognosis		(33)
	hsa_circ_100338	Up	Oncogene	SNX27	Up			Prognosis	HUVECs	(34)
	hsa_circ_0051443	Down	TS	TRAPPC6A	Up	miR-331-3p	CeRNA	Diagnostic	BAK1	(35)
	has_circ_0025129	Up	Oncogene	TNFRSF1A	Down	miR-34a	CeRNA	Prognosis	USP7	(36)
	circPTGR1^#^	Up	Oncogene	PTGR1	Down^*^	miR449a	CeRNA	Prognosis	MET	(37)
	hsa_circ_0001946	Up	Oncogene	CDR1	Down	miR-1270	CeRNA	Upregulated AFP	AFP	(38)
	hsa_circ_100284	Up	Oncogene	GCLM	Up	miR-217	CeRNA	Carcinogenesis by arsenite	EZH2	(39)
Colorectal Cancer	hsa_circ_0000677	Up	Oncogene	ABCC1	Up			Prognosis	Wnt/catenin	(40)
	hsa_circ_0010522	Up	Oncogene	RAP1GAP	Down^*^	miR-133a	CeRNA	Prognosis	GEF-H1/RhoA	(41)
	hsa_circ_0101802	Up	Oncogene	PNN	Up	miR-6833-3p/let-7i-3p/miR-1301-3p	CeRNA	Diagnostic		(42)
	hsa_circ_0005963	Up	Oncogene	TMEM128	Down^*^	miR-122	CeRNA	Resistance to oxaliplatin	PKM2	(43)
	hsa_circ_0069313	Up	Oncogene	PACRGL	Up	miR-142-3p/miR-506-3p	CeRNA	Prognosis	TGF-β1	(44)
	hsa_circ_0008558	Up	Oncogene	LONP2	Up	miR-17	CeRNA	Prognosis		(45)
	hsa-circ-0004771	Up	Oncogene	NRIP1	Down			Diagnostic		(46)
	hsa_circ_0005100	Up	Oncogene	FMN2	Down	miR-1182		Prognosis	hTERT	(47)
	hsa_circ_0000338	Down	TS	FCHSD2	Down^*^			FOLFOX-resistance		(48)
	hsa_circ_0067835	Up	Oncogene	IFT80	Up	miR-1236-3p	CeRNA	Prognosis	HOXB7	(49)
Pancreatic Cancer	hsa_circ_0036627	Up	Oncogene	PDE8A	Down	miR-338	CeRNA	Prognosis	MACC1/MET	(50)
	hsa_circ_0087502	Up	Oncogene	IARS	Up^*^	miR-122	CeRNA	Prognosis	ZO-1, RhoA, RhoA-GTP	(51)

(*) Statistically not significant. ^#^: In Ref (37), hsa_circ_0008043, hsa_circ_0003731, and hsa_circ_0088030 were all transcribed from the same gene (prostaglandin reductase 1, PTGR1) and were therefore collectively named circPTGR1; Up, Upregulated; Down, Downregulated; TS, Tumor suppressor; CeRNA, competitive endogenous RNAs; Ref., References.

### Expression of the Host Gene in Digestive System Tumors

TCGA data showed the expression profile of host genes of exosomal circRNAs in corresponding digestive system tumors. Compared with adjacent tissues, we found that the expression of the host gene CDR1 of hsa_circ_0001946 was downregulated in esophageal cancer; however, the difference was not statistically significant (**Table S1**). In gastric cancer, six and one host genes were upregulated and downregulated, respectively. Among them, RAB3A interacting protein (RAB3IP), Ran GTPase-activating protein 1 (RanGAP1), and KIAA1244 exhibited a fold change >2, indicating significantly high expression in gastric cancer (**Table S1**). The expression of Rap1 GTPase activating protein (RAP1GAP) was downregulated in gastric cancer, but the difference was not statistically significant (**Table S1**). In hepatocellular carcinoma, eight and four host genes were upregulated and downregulated, respectively. Among them, ubiquitin-like with PHD and ring finger domains 1 (UHRF1) and sorting nexin 27 (SNX27) with a fold change >2, revealing significantly high expression in hepatocellular carcinoma (**Table S1**). Three of the four downregulated host genes were 0.5 < fold change < 1, while the expression of prostaglandin reduce 1 (PTGR1) was downregulated in hepatocellular carcinoma, but the difference was not statistically significant (**Table S1**). In colorectal cancer, five and five host genes were upregulated and downregulated. Among them, the ATP-binding cassette subfamily C member 1 (ABCC1) and formin 2 (FMN2) showed fold change >2 and <0.5, respectively (**Table S1**). Five downregulated host genes were 0.5 < fold change < 1 or had no significant difference in the expression. In pancreatic cancer, there was no significant difference in the expression of the host genes of the two exosomes circRNAs compared with the adjacent tissues (**Table S1**). In general, in digestive system tumors, some exosomal circRNAs and their host genes exhibited similar expression patterns, whereas others showed opposite expression patterns (**Table 1**).

### Sponging of miRNAs and Their Target Genes in Digestive System Tumors

As mentioned earlier, 20 molecules of the 32 exosomal circRNA sponged miRNAs, adsorbing a total of 23 target miRNAs. We used the miRTargetLink Human online tool to predict the target genes of these 23 miRNAs and obtained 6,570 molecules: gastric cancer (1,741 target genes), hepatocellular carcinoma (1,795 target genes), colorectal cancer (2,704 target genes), and pancreatic cancer (330 target genes) (**Table S2**). We used two different methods to analyze these miRNAs and target genes.

**Figure 2:**
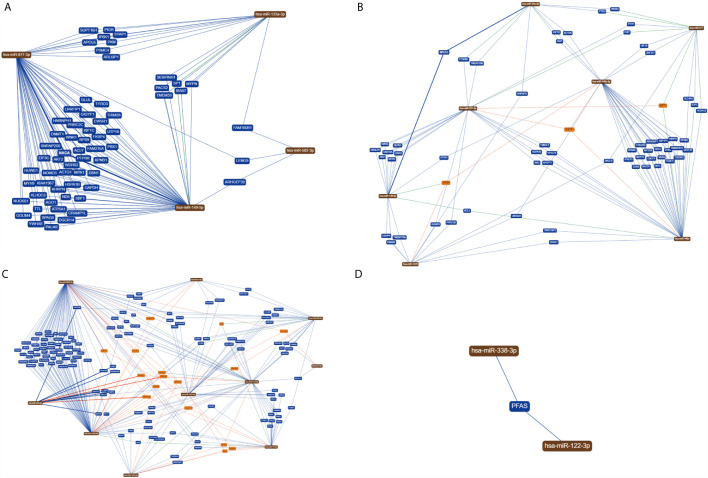
MiRNAs with the same target genes. **(A)** In gastric cancer, 63 target genes are regulated by at least two miRNAs; **(B)** in hepatocellular carcinoma, 60 target genes are regulated by at least two miRNAs; **(C)** in colorectal cancer, 146 target genes are regulated by at least two miRNAs; **(D)** In pancreatic cancer, only one target gene is regulated by two miRNAs at the same time.

Method 1: In the target genes of each type of digestive system tumors, we searched for genes that are regulated by at least two miRNAs, and performed GO and KEGG analyses on those. The results revealed 63, 60, 146, and one target gene in gastric cancer, hepatocellular carcinoma, colorectal cancer, and pancreatic cancer, respectively (**Figure 2** and **Table S3**).

Method 2: The data obtained from the TCGA database showed that, among the 6,570 target genes, only 1,146 target genes exhibited significant differential expression of ≥ 2 fold change (761 genes) or ≤ 0.5 fold change (385 genes): gastric cancer (278 genes), hepatocellular carcinoma (329 genes), colorectal cancer (535 genes), and pancreatic cancer (four genes) (**Table S6**). Subsequently, we performed functional enrichment analysis on all 1,146 target genes differentially expressed in digestive system tumors.

Intersection of the target genes obtained by two different methods in digestive system tumors, then we obtained ten, 11, 28 target genes in gastric cancer (NUCKS1, ACLY, SPAG5, EIF3C, TRIM28, TTL, PALM2, DNMT1, WDR62, UTP18), hepatocellular carcinoma (CENPN, SEPN1, UNC13A, FHIT, HSPA1B, TMEM120B, MAZ, NR4A2, E2F3, SLC4A2, BACH2), and colorectal cancer (CPEB3, PVRL4, CCL16, PPAP2B, PITPNM3, SLC25A32, SEMA3E, MYOCD, DCLK3, EVC2, KLF2, PLA2G16, LPP, C1orf115, CMKLR1, EMCN, PALM2, CENPJ, KCNK5, HMGA1, RANGAP1, TMEM41A, CDCA4, HMGB1, PNO1, PANK3, CD1D, RCAN1), respectively, and in pancreatic cancer, there were no same target gene in the intersection (**Figure S1**).

### Functional Enrichment of Target Genes

**Figure 3:**
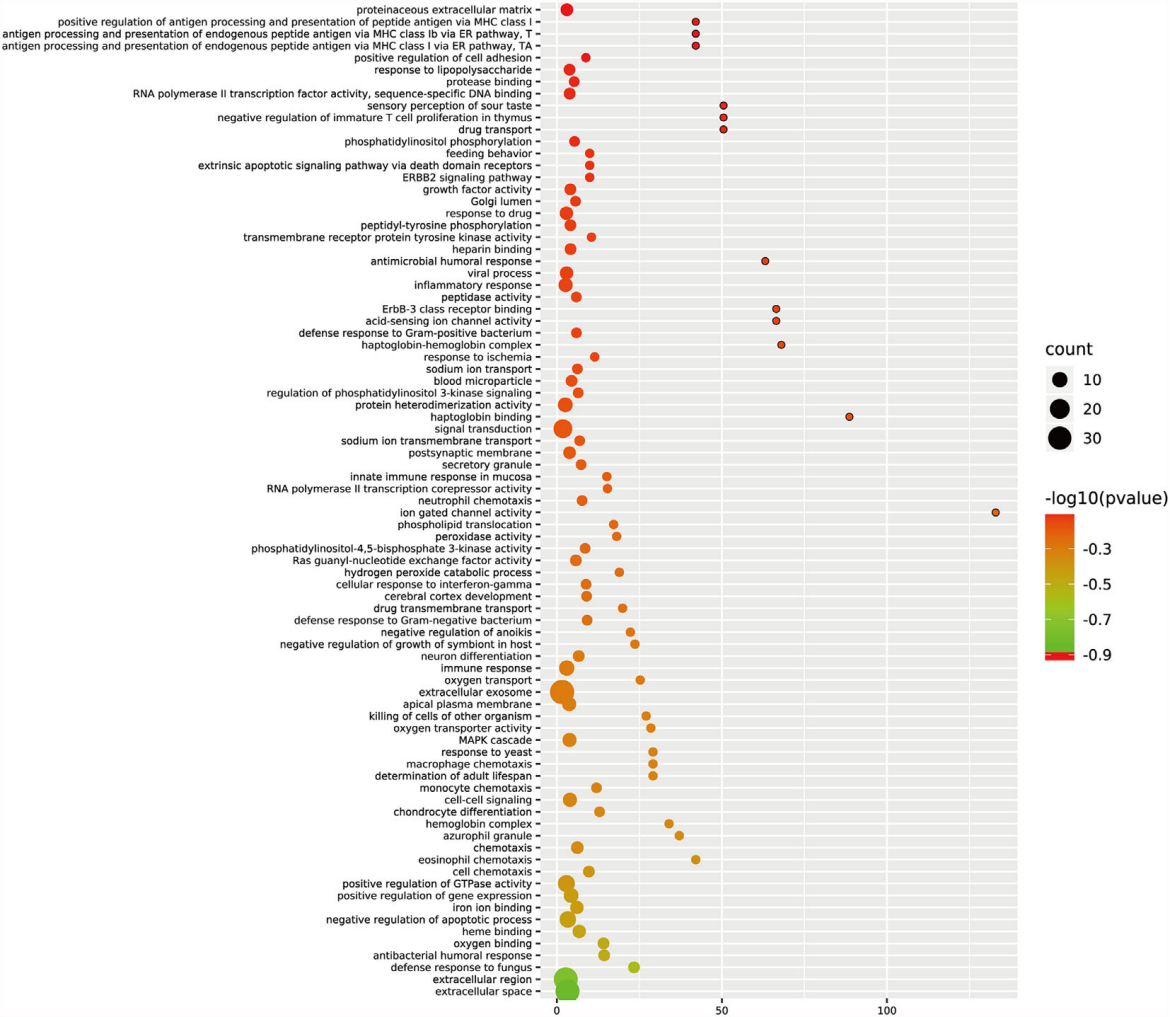
GO analysis of target genes in digestive system tumors. 270 target genes regulated by at least two miRNAs were involved in 83 cancer-related pathways. P < 0.05.

**Figure 4:**
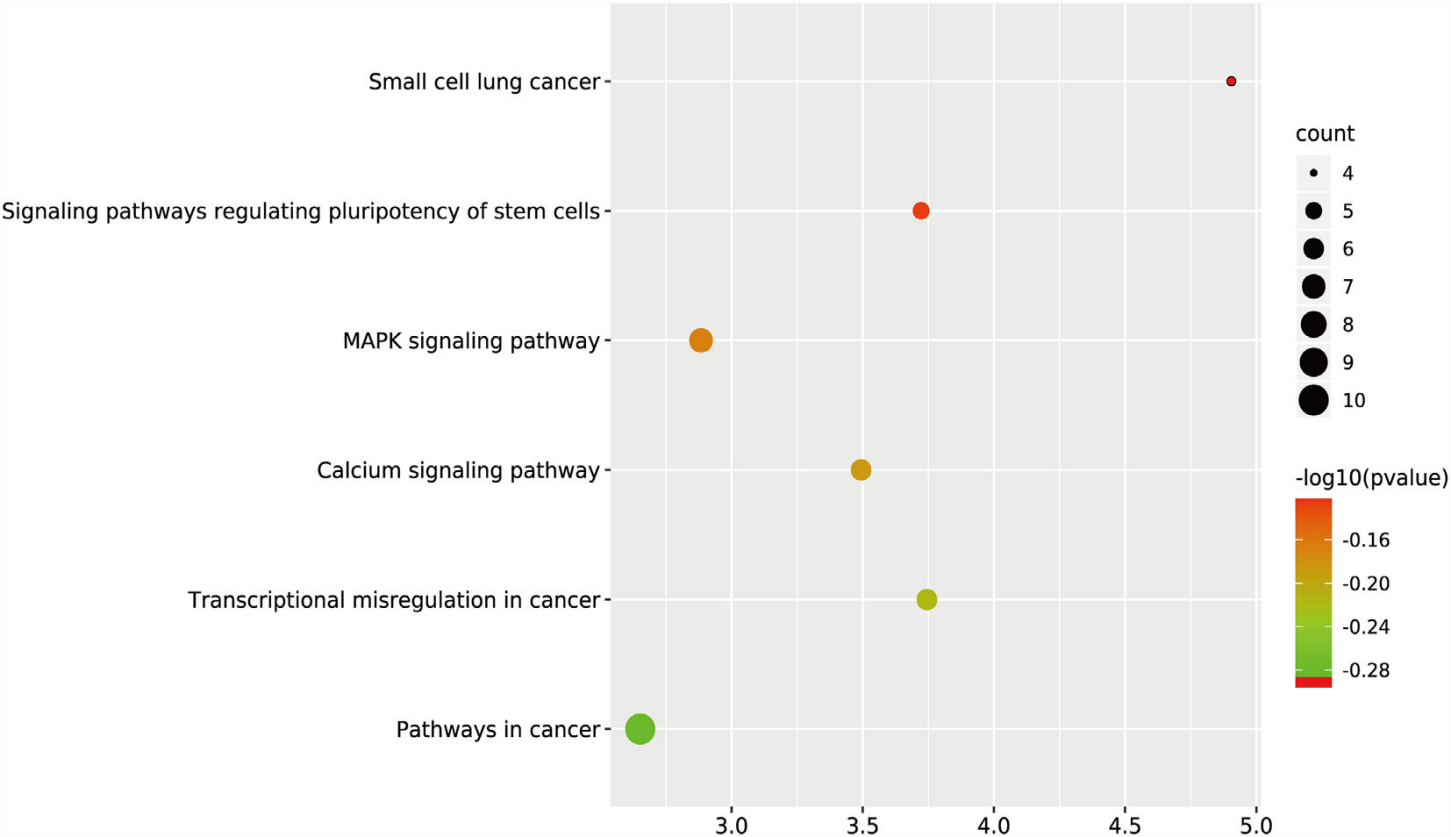
KEGG analysis of target genes in digestive system tumors. 270 target genes regulated by at least two miRNAs were involved in six KEGG pathways. P < 0.05.

GO and KEGG analyses were performed on the 270 target genes obtained through Method 1. The former showed that these genes were involved in 127 cancer-related functions, and the p-values of 83 cancer-related functions were <0.05 (**Table S4** and **Figure 3**). The latter showed that they were involved in 12 KEGG pathways, and the p-values of six KEGG pathways were <0.05 (**Table S5** and **Figure 4**).

**Figure 5:**
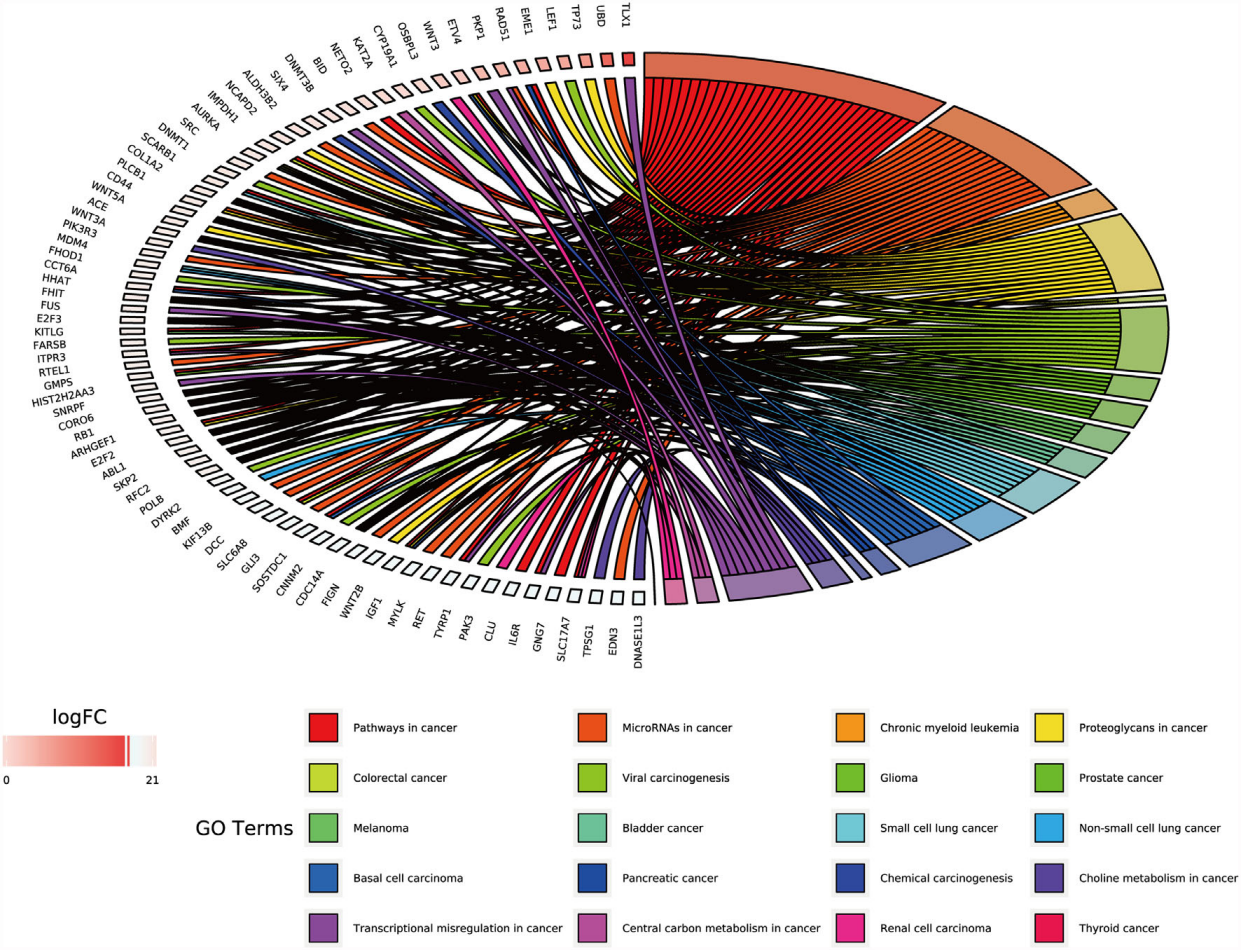
GO chord of target genes in digestive system tumors. 78 target genes differentially expressed in tumors of the digestive system were deeply involved in 20 cancer-related pathways. P < 0.05.

Functional enrichment analysis of the 1,146 differentially expressed target genes identified through Method 2 revealed that 78 genes related to other tumors were involved in 20 cancer-related pathways (e.g., chronic lymphocytic leukemia, glioma, prostate cancer, melanoma, bladder cancer, small cell lung cancer, non-small cell lung cancer, basal cell carcinoma, renal cell carcinoma, thyroid cancer, etc.) (**Table S7** and **Figure 5**).

### CircRNA-miRNA-mRNA Network in Digestive System Tumors

**Figure 6:**
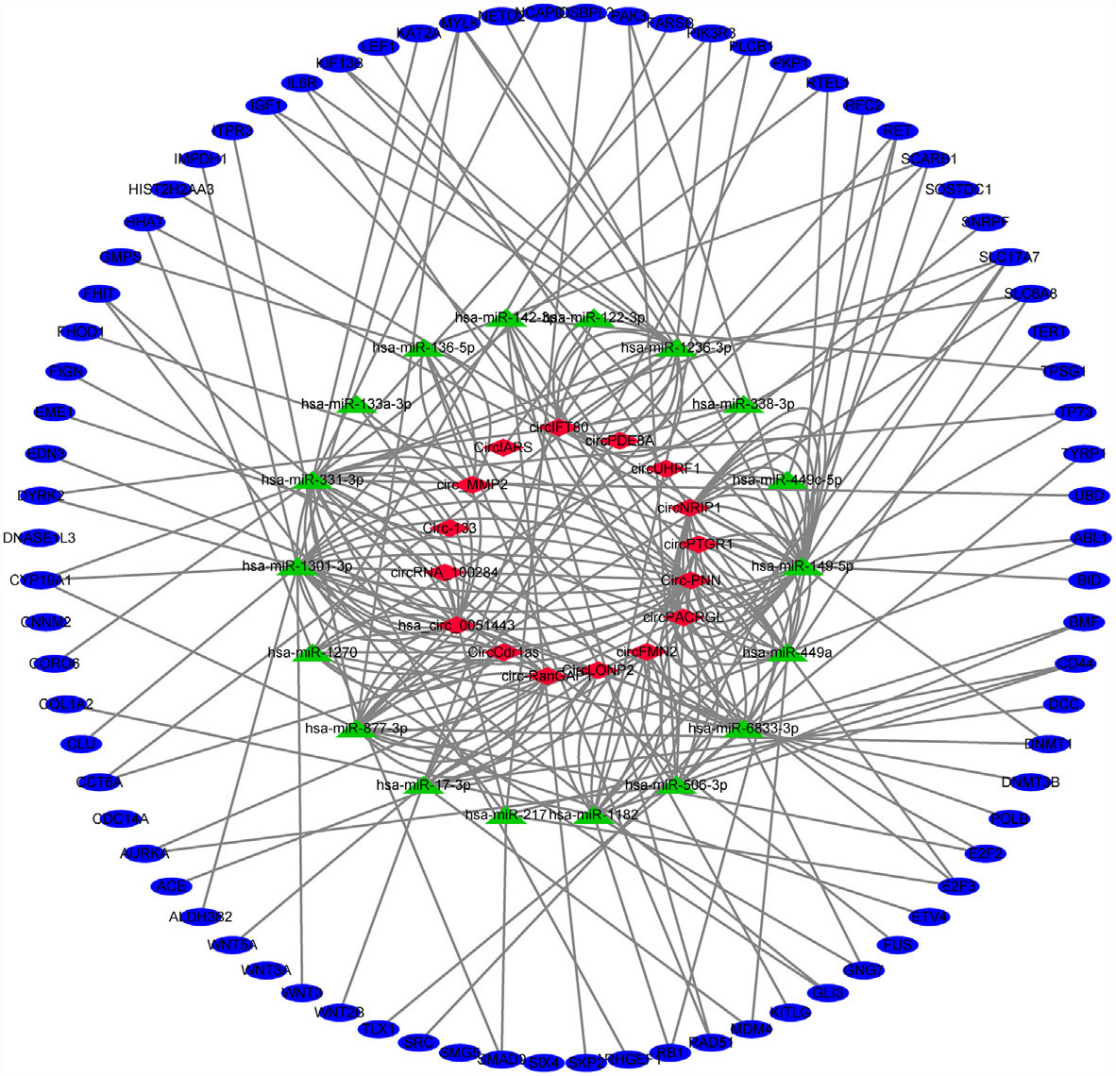
Exosomal circRNA-miRNA-mRNA network in digestive system tumors. 16 exosomal circRNAs closely related to digestive system tumors, which regulated 78 target genes through sponging of 18 miRNAs.

Finally, based on the previous analysis, we obtained 16 exosomal circRNAs closely related to digestive system tumors, which regulated 78 target genes through sponging of 18 miRNAs (**Table S7** and **Figure 6**). Both circ-RanGAP1 and its host gene were highly expressed in gastric cancer. Exosomes circ-RanGAP1 regulated 10 target genes (DNA methyltransferase 1 [DNMT1], E2F transcription factor 2 [E2F2], ETS variant transcription factor 4 [ETV4], cytochrome P450 family 19 subfamily A member 1 [CYP19A1], inositol 1,4,5-trisphosphate receptor type 3 [ITPR3], myosin light chain kinase [MYLK], ret proto-oncogene [RET], scavenger receptor class B member 1 [SCARB1], small nuclear ribonucleoprotein polypeptide F [SNRPF], solute carrier family 6 member 8 [SLC6A8]) through sponging hsa-miR-877-3p to promote the progression of gastric cancer (**Table 2**). Among the 10 target genes, the expression of MYLK, RET and SLC6A8 was down regulated in gastric cancer, while the expression of the other 7 genes was up-regulated in gastric cancer (**Table S6**). Both circUHRF1 and its host gene were highly expressed in hepatocellular carcinoma. The exosomal circUHRF1 regulated the expression of RET by sponging hsa-miR-449c-5p to promote the progression of hepatocellular carcinoma (**Table 2**). In colorectal cancer, circFMN2 was upregulated in, whereas its host gene was downregulated. Exosomes circFMN2 upregulated the expression of seven target genes (CD44, G protein subunit gamma 7 [GNG7], RB transcriptional corepressor 1 [RB1], collagen type I alpha 2 chain [COL1A2], phospholipase C beta 1 [PLCB1], solute carrier family 17 member 7 [SLC17A7], telomerase reverse transcriptase [TERT]) by binding miR-1182, thereby promoting the occurrence and development of colorectal cancer (**Table 2**).

**Table 2:** The main competing endogenous RNA pathways in three digestive system tumors.

Type of Cancer	CircRNA	Mode	MiRNA	Target Gene
Gastric cancer	circ-RanGAP1	1	hsa-miR-877-3p	DNMT1, E2F2, ETV4, CYP19A1, ITPR3, MYLK, RET, SCARB1, SNRPF, SLC6A8
Hepatocellular Cancer	circUHRF1	1	hsa-miR-449c-5p	RET
Colorectal Cancer	circFMN2	2	hsa-miR-1182	CD44, GNG7, RB1, COL1A2, PLCB1, SLC17A7, TERT

Mode 1 means the expression pattern of circRNA and its host gene is the same in the tumor. Mode 2 means the expression pattern of circRNA and its host gene is opposite in the tumor.

## Discussion

Exosomes are extracellular microvesicles with an average diameter of 100 nm and a double-layer liposome membrane (9). The biogenesis of exosomes is similar to endocytosis: the cell membrane is invaded to encapsulate proteins, non-coding RNA, lipids, etc. This forms intraluminal vesicles, and the luminal vesicles gradually form mature multivesicular bodies and are secreted as the exosomes (52). Exosomes secreted by cells can exist in a variety of body fluids (e.g., serum, plasma, saliva, urine, lymph, etc.), and are transported to distant tissue and cells to exert a wide range of regulatory effects (53). The substances in exosomes are diverse. Data (http://www.exocarta.org) have shown that 3,408 types of mRNA, 2,838 kinds of miRNA, 9,769 kinds of proteins, and 1,116 kinds of lipids are present in exosomes (54). CircRNA is another large type of non-coding RNA in exosomes, which is widespread in eukaryotes (55). This special molecule is formed by reverse splicing, which regulates the expression of the target protein by competitively binding miRNA, thereby affecting multiple signal transduction pathways and regulating multiple biological processes (56). It has been demonstrated that various exosomal circRNAs are involved in the regulation of malignant tumor proliferation, migration, invasion, and drug resistance. CircWHSC1 promotes the progression of ovarian cancer by competitively binding miR-1182 and miR-145 (57). CircSMARCA5 affects the malignant behavior of glioma by regulating serine and arginine rich splicing factor 1 (SRSF1)/SRSF3/PTB (57). Our research focused on tumors of the digestive system. We collected 32 exosomal circRNAs that were highly related to them, observed and predicted their sponging of miRNAs and target genes, analyzed the functions of these target genes and possible signal pathways involved in these processes. Moreover, we discussed their potential usefulness as tumor markers, as well as diagnostic and treatment targets.

RAB3IP, RanGAP1, and KIAA1244 are host genes of exosomal circRNA which exhibited more than two-fold differences in gastric cancer (**Table S1**). Although the fold change shown for RanGAP1 was not the highest, its homologous circRNA was the only molecule among the three circRNAs that has been proven to bind miRNA (26). Circ-RanGAP1 regulated vascular endothelial growth factor A (VEGFA) by targeting miR-877-3p, thereby promoting the metastasis and invasion of gastric cancer (26). At the same time, circ-RanGAP1/miR-877-3p may have 10 binding sites, such as DNMT1 in gastric cancer (**Table 2**). These proteins were closely related to multiple signaling pathways, such as cysteine and methionine metabolism, arachidonic acid metabolism, linoleic acid, cell cycle, etc. (**Table S7**). UHRF1 and SNX27 were host genes of exosomal circRNA that showed more than two-fold difference in expression in hepatocellular carcinoma (**Table S1**). Compared with normal liver tissues, the expression of UHRF1 in liver cancer was upregulated by 17.73-fold. Its homologous molecule circUHRF1 induced the depletion of natural killer T cells by targeting miR-449c-5p, thereby participating in the development of drug resistance by liver cancer cells (30). RET was a possible target protein of this process, and may be involved in the regulation of multiple signal pathways, such as peroxisome proliferator activated receptor (PPAR), PI3K-Akt, thyroid hormone, etc. (**Table S7**). ABCC1 and FMN2 were host genes of exosomal circRNA that were differentially expressed by more than two-fold in colorectal cancer (**Table S1**). Their homologous molecule hsa_circ_0000677 promoted the occurrence of colorectal cancer by directly regulating the Wnt signaling pathway, while circFMN2 promoted the proliferation of colorectal cancer by regulating miR-1182/hTERT (40, 47). CircFMN2/miR-1182 may also regulate the progression of colorectal cancer by targeting CD44, GNG7, RB1, COL1A2, PLCB1, SLC17A7, and TERT (**Table 2**). These target genes were widely involved in the regulation of extracellular matrix-receptor interaction, Ras, chemokine, PI3K-Akt, and other signaling pathways (**Table S7**). The function of these target genes in tumors warrants further experimental verification.

Interestingly, we found that circ-RanGAP1 and its host gene in gastric cancer, circUHRF1, as well as its host gene in hepatocellular carcinoma exhibited the same expression pattern. In contrast, circFMN2 and its host gene showed opposite patterns in colorectal cancer. CircRNA can be as a regulatory factor for host gene transcription and expression at both the transcription and post-transcription levels (58). However, currently, there are few studies on the interaction between circRNAs and their host genes, and the detailed mechanism involved in this process warrants further investigation.

We used two methods to screen the target genes, and performed functional enrichment analysis on the two screening results, respectively. The results of the miRNA-mRNA network analysis (**Figure 2**) and their functional enrichment analysis (**Figures 3** and **4**) in various digestive system tumors showed that 270 target genes were regulated by at least two miRNAs, which were involved in 83 cancer-related pathways and six KEGG pathways. Among them, the cancer-related functions with the most enriched genes (count ≥10) were extracellular space, extracellular region, extracellular exosome, negative regulation of the apoptotic process, positive regulation of GTPase activity, immune response, and signal transduction. The most enriched genes in the KEGG pathway were also “pathways in cancer”. The functional enrichment analysis of 1,146 target genes differentially expressed in tumors of the digestive system showed that 78 target genes were deeply involved in 20 cancer-related pathways (**Figure 5**).

The focus of the two screening methods is different. Method 1 screened the target genes regulated by at least two miRNAs in digestive system tumors. Most of these target genes are genes that play a key role in cell proliferation and metabolism, and they form a complex signal transduction network with a variety of miRNAs to regulate cell behavior. Phosphatase and tensin homologue (PTEN), as inhibitors of the phosphatidylinositol 3-kinase/Akt/mammalian target of rapamycin (PI3K/Akt/mTOR) pathway, is down-regulated by miR−29b and miR−301 in breast cancer, while it is regulated by miR−106b~25 and miR−22 in prostate cancer, which enhances the activity of the PI3K/Akt/mTOR pathway, thereby promoting tumor cell proliferation and inhibiting apoptosis (59). Similar to PTEN, the 270 target genes obtained by method 1 are all negatively regulated by multiple miRNAs, and construct a huge regulatory network in digestive system tumors, affecting the proliferation, invasion, migration, and other malignant phenotype of gastric cancer, hepatocellular carcinoma, and colorectal cancer, and pancreatic cancer. Method 2 screened out 1146 target genes that were significantly differentially expressed in four digestive system tumors. The differential expression of key genes in cancer and adjacent tissues is often the basis for changes in cell function (60). Several researches on the mechanism of tumors explore after discovering differentially expressed genes through microarray or sequencing (60, 61). These two methods only focus on a certain characteristic of the target gene in digestive system tumors, and the two complement each other. **Figure S1** shown the intersection of target genes obtained by two methods in gastric cancer (ten genes), hepatocellular carcinoma (11 genes), colorectal cancer (28 genes), and pancreatic cancer (zero genes). These 49 genes are in four digestive system tumors are significantly differentially expressed, and are regulated by at least two miRNAs. Therefore, they are more likely to play an irreplaceable role in digestive system tumors.

CircRNA as a miRNA sponge regulates tumor progression through the competing endogenous RNA mechanism. Non-coding RNA in exosomal exerts a wider range of effects to regulate tumors by promoting multiple-signal communication between tumor cells and the tumor microenvironment (including cancer related fibroblasts, tumor stem cells, macrophages, lymphocytes, mesenchymal stem cells etc) (13). Deepak Nagrath et al. found that mir-22, let7a and mir-125b in cancer-related fibroblast-derived exosomes change the energy metabolism of prostate cancer cells by inhibiting oxidative phosphorylation, which may be regulated by circRNA (62). Hong Zhao et al. found that hsa_circ_0000677 in exosomal activates Wnt signal pathway by mediating β-catenin into the nucleus, thereby significantly enhancing the metastatic ability and cell stemness of colorectal cancer (40). It has been reported that miR-503-3p in exosomes derived from bone marrow mesenchymal stem cells can increase the content of cancer stem cells in colorectal cancer, thereby promoting the growth of colorectal cancer (63). In addition to acting as competitive endogenous RNA, recent studies have found that circRNA with internal ribosome entry site (IRES) can bind to ribosomes and encode short peptides and proteins, thus directly regulating the function of tumor cells (64). Last but not least, as transcription factors or protein scaffolds, some circRNA interact directly with proteins to regulate the biological behavior of tumor cells (65).

**Figure 7:**
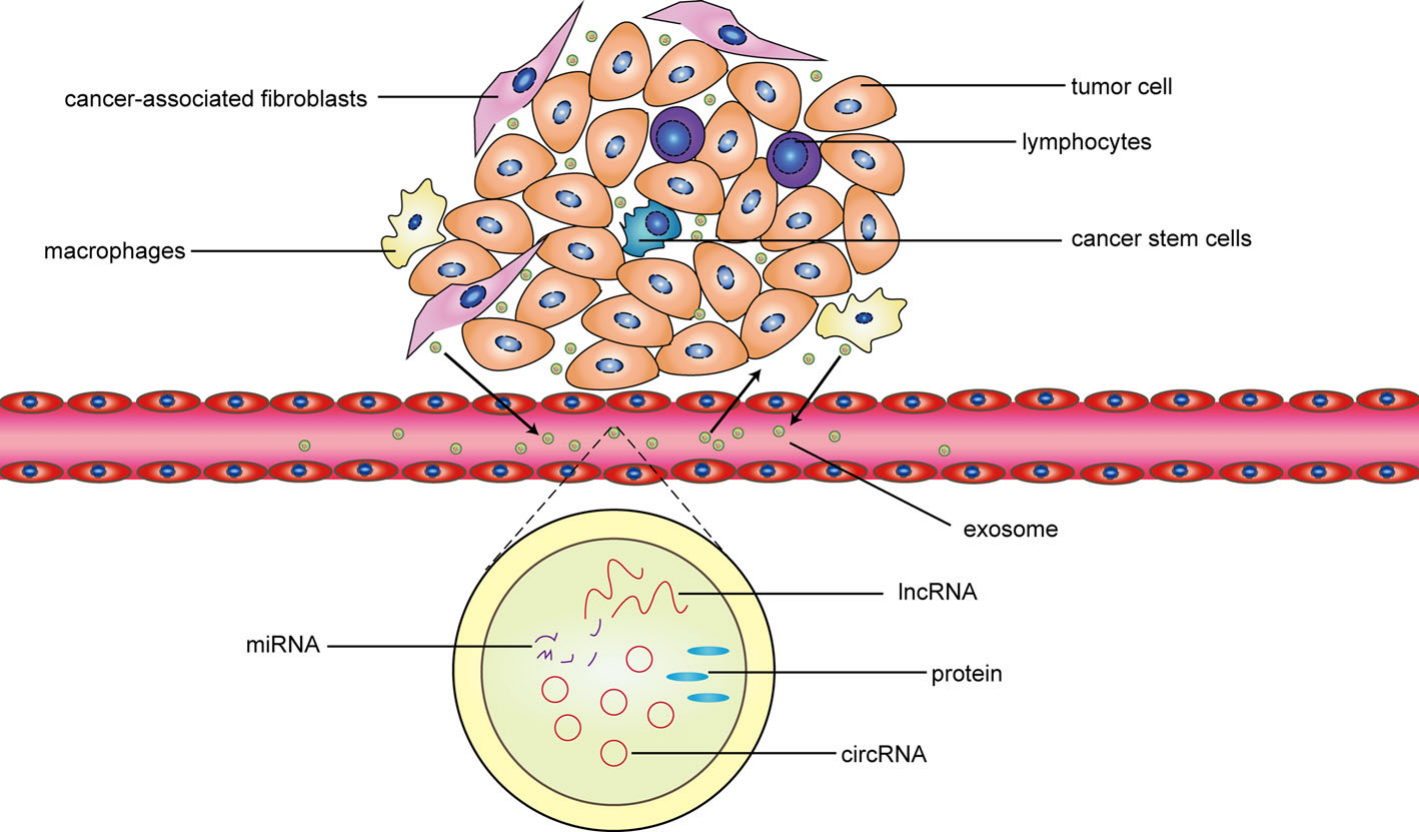
The interaction between exosomes and the tumor microenvironment. In general, cancer-related fibroblasts, cancer stem cells, macrophages and lymphocytes are important components of the tumor microenvironment. The exosomes released by the four types cells promote tumor proliferation, invasion, migration, drug resistance, and other malignant phenotype through the circRNA-miRNA-protein or lncRNA-miRNA-protein mechanism. Simultaneously, these four types cells are regulated by some exosomes released by the tumor cell. In addition, these exosomes not only act on adjacent tissue cells, but also be transported to the distal through blood to take effect.

Exosomes also have a profound impact on immune cells in the tumor microenvironment. The exosomes secreted by tumors can promote the transformation of macrophages into the M2 type, increase the activity of regulatory T cells, and inhibit natural killer cell toxicity, thereby forming immunity inhibition. Simultaneously, some exosomes produced by immune cells inhibit the maturation of antigen-presenting cells and the secretion of anti-tumor factors, and participate in the occurrence and development of tumors (66–68). PengFei Zhang et al. confirmed that circUHRF1 inhibits the activation of natural killer cells and the release of cytokines (IFN-γ and TNF-α) through sponge miR-449c-5p in hepatocellular carcinoma, which induced resistance to anti-PD-1 therapy in patients with hepatocellular carcinoma (30). In gastric cancer, exosomal ciRS-133 promotes white adipose tissue browning by inhibiting miR-133 and activating PRDM16, thereby reducing the oxygen consumption of the gastric cancer microenvironment and promoting tumor growth (28). In short, exosomes non-coding RNA promote the malignant phenotype of tumors through the aforementioned processes, and inhibit the detection and killing tumors by immune cells, thereby participating in the occurrence and development of malignant tumors (**Figure 7**). Exosomes, as intercellular messengers, widely exist in various body fluids and are easy to be extracted, isolated and identified. The differential expression of non-coding RNA between tumor and normal tissues and cells provides an effective marker for clinical diagnosis and treatment of diseases (69). Therefore, exosomal non- coding RNA has great potential in future cancer research. However, in the studies of exosomal non-coding RNA regulating tumors, we noticed that the research on exosomal miRNA and long non-coding RNA has been abundant, while the related research on exosomal circRNA is extremely limited. The mechanism of exosomal circRNA regulating tumor cells and tumor microenvironment still needs further explore.

Our research and previous literature have shown that exosomal circRNA plays a significant role in the progression of a various digestive system tumors, particularly gastric cancer, hepatocellular carcinoma, and colorectal cancer. Bioinformatics research is helpful for screening molecules and identifying valuable intervention targets. However, this approach is characterized by limitations and certain biases compared with biological experiments. Thus, our results warrant further experimental verification.

## Conclusion

In general, the expression of exosomal circRNA is stable and easily detectable in body fluids. Circ-RanGAP1 in gastric cancer, circUHRF1 in hepatocellular carcinoma, and circFMN2 in colorectal cancer may be useful as tumor markers, as well as diagnostic and treatment targets. These factors may improve early diagnosis and prognosis of these three gastrointestinal malignancies.

## Data Availability Statement

The original contributions presented in the study are included in the article/**Supplementary Material**. Further inquiries can be directed to the corresponding authors.

## Author Contributions

Conceptualization: HW and XZ. Formal analysis: HW and YZhe. Funding Acquisition: YZho. Writing—original draft: HW. Writing—review and editing: HW and YW. All authors contributed to the article and approved the submitted version.

## Funding

This research was supported by the National Natural Science Foundation of China (71964021), National Key R&D Program of China (2016YFC1302201 and 2017YFC0908302), the University Scientific Research Project of Gansu Province (2018B-011), the Fundamental Research Funds for the Central Universities (lzujbky-2020-kb16).

## Conflict of Interest

The authors declare that the research was conducted in the absence of any commercial or financial relationships that could be construed as a potential conflict of interest.

## Supplementary Material

Supplementary Figure 1 Venny map for the outcomes of two different gene screening methods in various digestive system tumors. Method 1 searched for target genes that were regulated by at least two miRNAs. Method 2 obtained target genes that significant differential expression of ≥ two-fold change. **(A)** Ten same target genes obtained from method 1 and method 2 in gastric cancer (NUCKS1, ACLY, SPAG5, EIF3C, TRIM28, TTL, PALM2, DNMT1, WDR62, UTP18); **(B)** 11 same target genes obtained from method 1 and method 2 in hepatocellular carcinoma (CENPN, SEPN1, UNC13A, FHIT, HSPA1B, TMEM120B, MAZ, NR4A2, E2F3, SLC4A2, BACH2); **(C)** 28 same target genes obtained from method 1 and method 2 in colorectal cancer (CPEB3, PVRL4, CCL16, PPAP2B, PITPNM3, SLC25A32, SEMA3E, MYOCD, DCLK3, EVC2, KLF2, PLA2G16, LPP, C1orf115, CMKLR1, EMCN, PALM2, CENPJ, KCNK5, HMGA1, RANGAP1, TMEM41A, CDCA4, HMGB1, PNO1, PANK3, CD1D, RCAN1); **(D)** there were no same target gene from method 1 and method 2 in pancreatic cancer.
