# Supplementary figures and images for: Exosomal circRNA in Digestive System Tumors: The Main Player or Coadjuvants?
Source: Front Oncol. 2021 Jun 24;11:614462. doi: 10.3389/fonc.2021.614462 (PMC8264426; doi:10.3389/fonc.2021.614462)

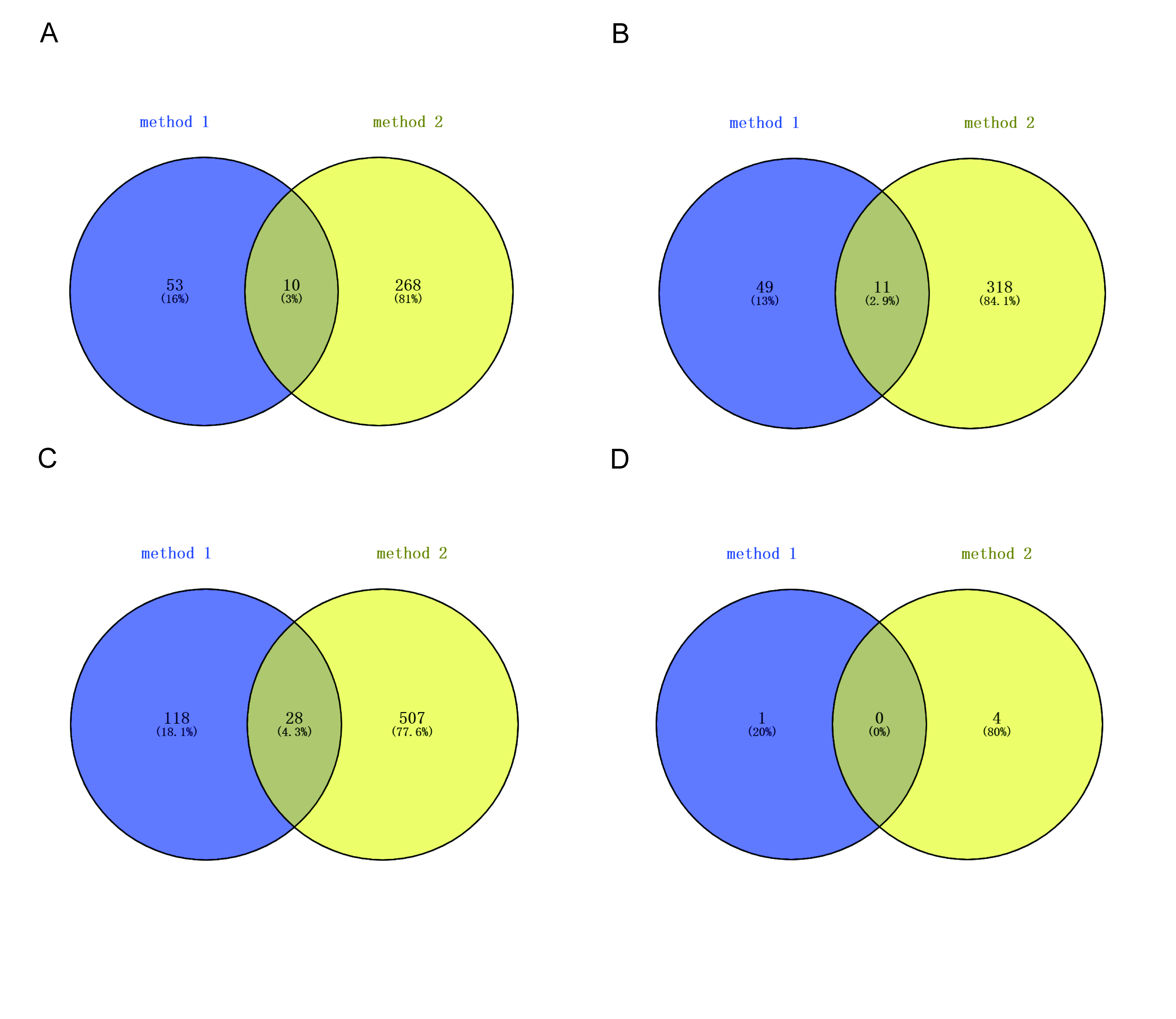

Supplement: Supplementary Figure 1 — Venny map for the outcomes of two different gene screening methods in various digestive system tumors. Method 1 searched for target genes that were regulated by at least two miRNAs. Method 2 obtained target genes that significant differential expression of ≥ two-fold change. (A) Ten same target genes obtained from method 1 and method 2 in gastric cancer (NUCKS1, ACLY, SPAG5, EIF3C, TRIM28, TTL, PALM2, DNMT1, WDR62, UTP18); (B) 11 same target genes obtained from method 1 and method 2 in hepatocellular carcinoma (CENPN, SEPN1, UNC13A, FHIT, HSPA1B, TMEM120B, MAZ, NR4A2, E2F3, SLC4A2, BACH2); (C) 28 same target genes obtained from method 1 and method 2 in colorectal cancer (CPEB3, PVRL4, CCL16, PPAP2B, PITPNM3, SLC25A32, SEMA3E, MYOCD, DCLK3, EVC2, KLF2, PLA2G16, LPP, C1orf115, CMKLR1, EMCN, PALM2, CENPJ, KCNK5, HMGA1, RANGAP1, TMEM41A, CDCA4, HMGB1, PNO1, PANK3, CD1D, RCAN1); (D) there were no same target gene from method 1 and method 2 in pancreatic cancer. [file Image_1.tif]
